# Analysis of Shared Genetic Regulatory Networks for Alzheimer's Disease and Epilepsy

**DOI:** 10.1155/2021/6692974

**Published:** 2021-10-14

**Authors:** Xiao-Dan Wang, Shuai Liu, Hui Lu, Yalin Guan, Hao Wu, Yong Ji

**Affiliations:** Department of Neurology, Tianjin Huanhu Hospital, Tianjin Key Laboratory of Cerebrovascular and Neurodegenerative Diseases, Tianjin Dementia Institute, Tianjin 300350, China

## Abstract

Alzheimer's disease (AD) and epilepsy are neurological disorders that affect a large cohort of people worldwide. Although both of the two diseases could be influenced by genetic factors, the shared genetic mechanism underlying the pathogenesis of them is still unclear. In this study, we aimed to identify the shared genetic networks and corresponding hub genes for AD and epilepsy. Firstly, the gene coexpression modules (GCMs) were constructed by weighted gene coexpression network analysis (WGCNA), and 16 GCMs were identified. Through further integration of GCMs, genome-wide association studies (GWASs), and expression quantitative trait loci (eQTLs), 4 shared GCMs of AD and epilepsy were identified. Functional enrichment analysis was performed to analyze the shared biological processes of these GCMs and explore the functional overlaps between these two diseases. The results showed that the genes in shared GCMs were significantly enriched in nervous system-related pathways, such as Alzheimer's disease and neuroactive ligand-receptor interaction pathways. Furthermore, the hub genes of AD- and epilepsy-associated GCMs were captured by weighted key driver analysis (wKDA), including *TRPC1*, *C2ORF40*, *NR3C1*, *KIAA0368*, *MMT00043109*, *STEAP1*, *MSX1*, *KL*, and *CLIC6*. The shared GCMs and hub genes might provide novel therapeutic targets for AD and epilepsy.

## 1. Introduction

Alzheimer's disease (AD) is a neurodegenerative disease characterized by memory difficulty, daily activity dysfunction, and cognitive decline, with neuropathological lesion and neuron loss in the brain [1–3]. This disease affects approximately 36 million people worldwide, and the number of AD patients is estimated to triple by 2050, along with the prolonged life expectancy of global population [4, 5]. Currently, the therapies of AD mainly focus on counterbalancing the neurotransmitter disturbance, and there have not been effective pharmacotherapeutic options for the prevention and treatment of AD yet [6, 7]. Another neurological disorder, epilepsy, is a common brain condition with an unprovoked seizure of high recurrence rate, which is defined epilepsy based on at least one of the following conditions: (1) two unprovoked seizures that occur more than 24 h apart and (2) diagnosis of an epilepsy syndrome [8, 9]. Epilepsy is known to be closely related to the psychosocial, neurobiological, and cognitive statuses and affects more than 70 million people worldwide [8, 10]. Despite the advances in antiepileptic drugs and surgeries, epilepsy treatments still confront some challenges, such as the resistance to medical treatment, underutilization of epilepsy surgery, and the gaps in epilepsy-related knowledge [11]. Moreover, the etiologies of AD and epilepsy are still unclear [12, 13].

It is believed that there is an association between AD and epilepsy [14]. Both AD and epilepsy are neurological diseases [15], and their incidence risks are proven to elevate with age. AD is known to influence more than 40% of the people over the age of 85 [16], and the incidence rate of epilepsy also remains high in patients over 50 years old and peaks at the age of 70 years [10]. In addition, accumulating evidence has proven that AD contributes to higher risk of epilepsy [17–19]. It is reckoned that 10-22% of AD patients have epilepsy [20]. Pathological alterations in entorhinal cortex, subiculum, and hippocampal field CA1 are observed in both temporal lobe epilepsy and AD [18, 21, 22]. Furthermore, both AD and epilepsy are associated with genetic factors. It has been proven that genetic factors play a pivotal role in the occurrence of AD. In nonfamilial AD, genetic factors have a predominant role and account for more than 60% of the cases; while in familial AD, the familial genes in AD are autosomal dominant, often with point mutations in presenilin 1, presenilin 2, and amyloid precursor protein [23]. *SCN1A* is recognized as an epilepsy-related gene, and its variants are related to inherited genetic epilepsy [24]. Rohena et al. have proposed *SNAP25* mutation as a risk factor for epilepsy, which could also lead to ataxia and seizure in animal model [25]. Scher et al. have reported *MTHFR* C677T variant as a potential genetic cause for epilepsy [26]. In addition, the shared genetic background of AD and epilepsy has also been explored. The presenilin 1 gene (*PSEN1*) mutations are demonstrated to be associated with AD and epilepsy. Epileptic seizures are recognized as the clinical phenotypes of PSEN1 AD, and PSEN1 AD is suggested to be considered as a genetic epilepsy syndrome according to the new International League Against Epilepsy nomenclature [27]. The genetic form of AD is characterized by aberrant amyloid-*β* and potentially related to seizures [14]. Toral-Rios et al. have proposed that the changes in GSK3*β* and the encoding genes of tau are the genetic factors that contribute to AD and temporal lobe epilepsy developments [28].

However, the treatment of epilepsy in patients with AD remains to be a challenge, because the patients are prone to be affected by drug interactions and adverse effects [20]. If there is a close relationship between epilepsy and AD, the therapeutic strategies may be shared. Therefore, the association between epilepsy and AD is a pivotal issue. In this research, we integrated the gene coexpression modules (GCMs), genome-wide association studies (GWASs), and expression quantitative trait loci (eQTLs) to identify the shared GCMs of AD and epilepsy. Then, the shared biological processes of the GCMs were investigated to analyze the functional overlaps between these two diseases through functional enrichment analysis. In addition, the hub genes of AD- and epilepsy-associated GCMs were captured by weighted key driver analysis (wKDA). The shared GCMs and hub genes may be novel therapeutic targets for both AD and epilepsy.

## 2. Materials and Methods

### 2.1. Study Population

The gene expression profiles of epilepsy and AD samples were obtained from the Gene Expression Omnibus (GEO, https://www.ncbi.nlm.nih.gov/geo/) database. The epilepsy samples (access number: GSE63808, platform: Illumina HumanHT-12 V3.0) included 129 epilepsy patients, and the AD samples (access number: GSE132903, platform: Illumina HumanHT-12 V4.0) consisted of 97 AD patients and 98 healthy controls. The genome-wide association study (GWAS) dataset of AD samples was obtained from Database of Genotypes and Phenotypes (dbGaP, https://www.ncbi.nlm.nih.gov/gap, access number: phs000219).

### 2.2. Data Preprocessing

Due to the difference in platforms of GSE63808 and GSE132903 datasets, the annotated genes were different. The removeBatchEffect function in limma package (http://www.bioconductor.org/packages/release/bioc/html/limma.html) [29] was used to remove the batch effects after obtaining the shared genes of the two platforms. Then, the two datasets were integrated into a matrix for further analysis.

### 2.3. Construction of Gene Coexpression Modules (GCMs)

The AD (case) and epilepsy expression data were used together for the GCM identification. The control samples in the GSE132903 dataset were not included in this analysis. We performed the weighted gene coexpression network analysis (WGCNA) by using WGCNA R package [30]. First, the similarity matrix was constructed by calculating the Pearson correlation coefficient between every two genes with the following equation: *S*_*ij*_ = ∣(1 + cor(*x*_*i*_ + *y*_*j*_))/2∣. Then, the similarity matrix was transformed to adjacency matrix with the equation of *α*_*ij*_ = |(1 + cor(*x*_*i*_ + *y*_*j*_))/2|^*β*^, and *β* represented the soft-threshold. The topological matrix was obtained based on the topological overlap measure (TOM) = (∑_*μ*≠*ij*_*α*_*iμ*_*α*_*μj*_ + *α*_*ij*_)/(min(∑_*μ*_*α*_*iμ*_ + ∑_*μ*_*α*_*jμ*_) + 1 − *α*_*ij*_).

Hierarchical clustering analysis was carried out using 1-TOM, the index which reflected the similarity between every two genes. The modules were identified with dynamic branch cutting method, and the closely interconnected genes were placed into the same module (minimum module size = 30). Module eigengene (ME), the representative gene of each module that reflected the whole expression level of corresponding module, was calculated using the equation of ME = princomp (*x*_*ij*_^*q*^).

### 2.4. Identification of Shared GCMs between AD and Epilepsy

The Marker Set Enrichment Analysis (MSEA) in Mergeomics [31] was adopted to identify the shared GCMs between AD and epilepsy, and the parameters that were used followed the default pipeline stablished by Shu et al. *P* value = 0.05 was used as the threshold for screening, and the filtered expression quantitative trait loci (eQTLs) were analyzed by MSEA. Three files were input: the summary results of GWAS, the eQTL information of single nucleotide polymorphisms (SNPs), and the GCM results of WGCNA.

### 2.5. Functional Enrichment Analysis

The KOBAS 3.0 (http://kobas.cbi.pku.edu.cn/index.php) was used to perform the functional enrichment analysis of genes. *P* < 0.05 was considered as the threshold to screen for significantly enriched Kyoto Encyclopedia of Genes and Genomes (KEGG) and Reactome pathways.

### 2.6. Identification of Hub Genes

Hub genes are defined as the genes with the highest degree (the number of genes connected to the hub) in the gene coexpression networks calculated by maximum neighborhood component (MNC) algorithm. The weighted key driver analysis (wKDA) in Mergeomics was used to identify the hub genes of each GCM, and the results were visualized by the Cytoscape software [32]. The above analyses were performed using the R software (version 3.5.2).

## 3. Results

### 3.1. A Total of 16 GCMs Were Identified for AD and Epilepsy Samples

**Figure 1:**
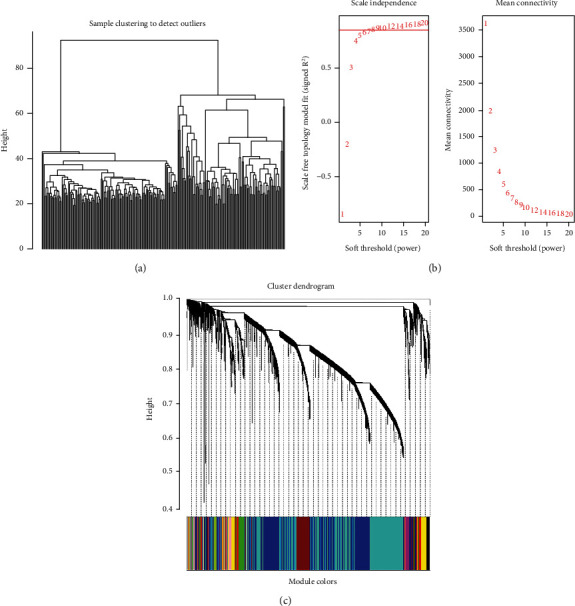
Construction of gene coexpression modules. (a) Clustering analysis of the samples showed there were no outlier samples. (b) The soft-threshold was selected as *β* = 8 to satisfy the criteria of scale free topology. (c) Gene dendrogram showed that 16 coexpression modules were identified. The gray module denoted the genes that could not be classified into any modules.

There were 22,614 common genes for the gene expression profiles of AD and epilepsy samples. After removal of batch effects and integration of matrix, 22,614 genes were obtained. Clustering analysis showed that there were no outlier samples (Figure 1(a)). The soft-threshold was selected as *β* = 8 to meet the criteria of scale free topology (the correlation coefficient between log(*k*) and log(*p*(*k*)) greater than 0.8) (Figure 1(b)) [33, 34].

After identification of the gene modules, the ME of each module was calculated. Then, clustering analysis was performed on the modules, and the highly correlated modules were merged (height = 0.25). As shown in Figure 1(c), 16 modules were obtained.

### 3.2. Shared GCMs between AD and Epilepsy

**Figure 2:**
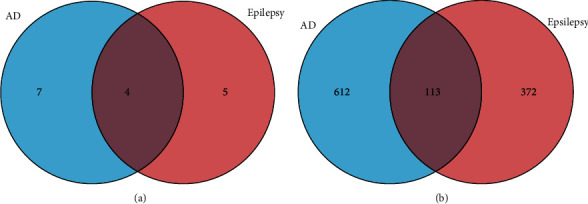
Venn diagrams of overlap in gene coexpression modules and pathways between AD and epilepsy. (a) Overlap in gene coexpression modules between AD and epilepsy. (b) Overlap in pathways between AD and epilepsy.

The identified 16 modules included AD-related modules, epilepsy-related modules, and modules associated with both AD and epilepsy. The shared GCMs between AD and epilepsy were identified by MSEA using the GCM information above and the GWAS data of AD. As shown in Figure 2(a), 11 modules were associated with AD and 9 with epilepsy. Among them, four modules (the brown, yellow, red, and midnightblue modules) were associated with both AD and epilepsy. Functional enrichment analysis of the genes in these 16 modules revealed that there were 725 pathways related to AD and 485 pathways related to epilepsy. Among them, 113 pathways were related to both AD and epilepsy (Figure 2(b)).

### 3.3. Shared Biological Processes of AD- and Epilepsy-Associated GCMs

**Figure 3:**
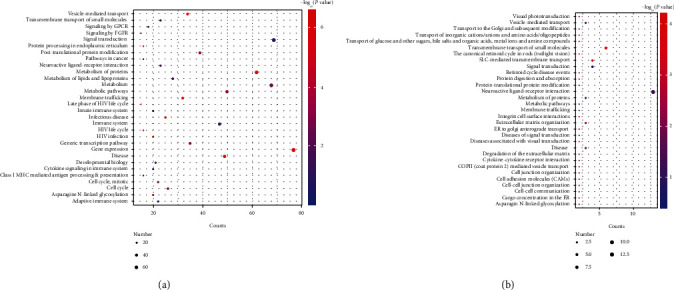
Function enrichment analysis of the genes in the (a) brown and (b) midnightblue modules. The vertical axis represented the pathways, and the color alteration of the dot from red to blue indicated the alteration of *P* value from large to small.

Functional enrichment analysis of the genes in the 4 shared modules between AD and epilepsy was carried out. The significantly enriched pathways in the brown, midnightblue, red, and yellow modules are shown in Figures 3(a) and 3(b) and Figure S1A and Figure S1B, respectively. There were 713 genes in brown module, 152 in midnightblue module, 287 in red module, and 486 in yellow module. In addition, the result of functional enrichment analysis of these four modules was provided in Table S1. It was found that the genes in the 4 shared GCMs were significantly enriched in nervous system-related pathways, such as AD and neuroactive ligand-receptor interaction, indicating that the genes in these GCMs played a key role in AD and epilepsy.

### 3.4. Hub Genes of AD- and Epilepsy-Associated GCMs

**Figure 4:**
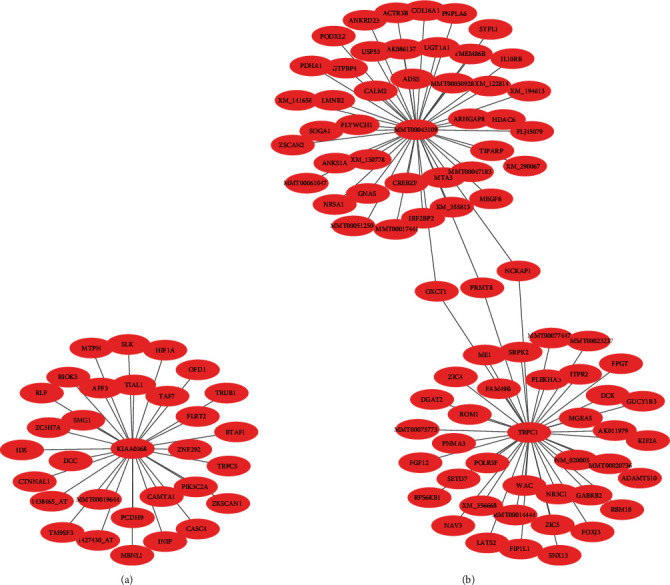
Identification of the hub genes for two of the 4 shared GCMs. (a) *KIAA0368* was the hub gene of the brown module. (b) *MMT00043109* and *TRPC1* were the hub genes of the red module.

The wKDA in Mergeomics was used to construct the gene expression networks for the 4 shared GCMs. Then, the hub genes were identified. As shown in Figure 4 and Figure S2, *KIAA0368* in the brown module, *MMT00043109* and *TRPC1* in the red module, *STEAP1*, *C2ORF40*, *MSX1*, *KL*, and *CLIC6* in the midnightblue module, and *NR3C1* in the yellow module exhibited the highest degree in each of the gene coexpression networks and were identified as the hub genes of each module.

## 4. Discussion

As neurological disorders, AD and epilepsy are considered to be interconnected with shared symptoms and etiologies [35]. In addition, genetic factors also play a crucial role in the pathogenesis of AD and epilepsy. In this research, we constructed 16 GCMs and identified 4 shared GCMs between AD and epilepsy by integrated analyses of GCMs, GWAS, and eQTLs. To analyze the functional overlaps between the two diseases, the shared biological processes of these GCMs were investigated by functional enrichment analysis. It revealed that the genes in the shared GCMs were significantly enriched in nervous system-related pathways, such as AD and neuroactive ligand-receptor interaction pathways. Furthermore, the hub genes of each AD- and epilepsy-associated GCM were captured, including *TRPC1*, *C2ORF40*, *NR3C1*, *KIAA0368*, *MMT00043109*, *STEAP1*, *MSX1*, *KL*, and *CLIC6*.

Transient receptor potential canonical 1 (*TRPC1*) encodes the TRPC1 protein, which is a crucial member of the TRPC proteins [36]. TRPC proteins are main gates of Ca^2+^ entry and participate in multiple biological processes, including transcription factor activation and cell proliferation [37]. As first reported in 1995, TRPC1 protein can interact with TRPC4 and TRPC5 to form TRPC1/4/5 channels, which are significantly implicated in epilepsy [38–40]. It is generally known that TRPC1/4 channels can lead to the excitotoxicity and epileptiform burst firing in the CA1 and the lateral septum, which are pathophysiological elements of epilepsy [41, 42]. Consequently, *TRPC1* is the potential target for the treatment of epilepsy. Previous study has suggested that calcium homeostasis dysfunction could occur in AD [43]. Due to the vital effect of *TRPC1* on Ca^2+^ entry, it is hypothesized that *TRPC1* is also probably associated with AD. A previous study confirmed that *TRPC1* played a protective role in neurodegeneration and neurotoxicity, which were typical features of AD, through regulation of Ca^2+^ influx [44–46]. Therefore, *TRPC1* may be a shared genetic risk factor for AD and epilepsy.

The orphan *C2ORF40* gene encodes Ecrg4, a preprotein precursor related to neural progenitor responsiveness of central nervous system injury [47, 48]. The expression of *C2ORF40* alters in some brain cells of AD patients, which is probably implicated in the neuroimmune response in AD *via* recruiting the microglia or infiltrating monocytes to the white matter [49], while in status epilepticus, the infiltrating monocytes could enhance the inflammation of brain and accelerate neural injury [50]. Based on these studies, we speculate that *C2ORF40* may also be involved in epilepsy by affecting the infiltrating monocytes.

*NR3C1* encodes glucocorticoid receptor (GR), a 94 kDa protein that belongs to the superfamily of nuclear hormone receptors [51]. GR plays an important role in neuron function by binding to glucocorticoid hormones [52]. In the mouse model of AD, the early downregulation of GR was observed, and the decreased GR level could be normalized by rosiglitazone, a potent agonist used for the cognitive function improvement for AD patients [53]. Brain-derived neurotrophic factor (*BDNF*), highly expressed in the brain, is a crucial regulator of neuron function, and the altered expression of *BDNF* is related to epilepsy, while GR shares several similarities with *BDNF* and is able to regulate *BDNF* expression level [52]. Therefore, it is proposed that the GR-encoding gene *NR3C1* may participate in the pathogenesis of epilepsy by regulating *BDNF*, and *NR3C1* is a possible shared gene for AD and epilepsy.

In conclusion, we identified 4 shared GCMs and 9 hub genes, including *TRPC1*, *C2ORF40*, *NR3C1*, *KIAA0368*, *MMT00043109*, *STEAP1*, *MSX1*, *KL*, and *CLIC6* through integrating analyses of genomics and genetics. The shared GCMs and hub genes should be helpful for unraveling the current difficulty of epilepsy treatment in AD and providing novel shared therapeutic strategies targeting both of these two diseases.

## Data Availability

All data generated or analyzed during this study are included in this published article.

## Supplementary Materials

Supplementary 1 Figure S1: function enrichment analysis of the genes in the red (A) and yellow modules (B). The vertical axis represented the pathways, and the color alteration of the dot from red to blue indicated the alteration of *P* value from large to small.

Supplementary 2 Figure S2: identification of the hub genes for another two of the 4 shared GCMs. (A) *STEAP1*, *C2ORF40*, *MSX1*, *KL*, and *CLIC6* were the hub genes of the midnightblue module. (B) *NR3C1* was the hub gene of the yellow module.

Supplementary 3 Table S1: functional enrichment analysis of these four modules.
