# Supplementary figures and images for: Analysis of Shared Genetic Regulatory Networks for Alzheimer's Disease and Epilepsy
Source: Biomed Res Int. 2021 Oct 14;2021:6692974. doi: 10.1155/2021/6692974 (PMC8538392; doi:10.1155/2021/6692974)

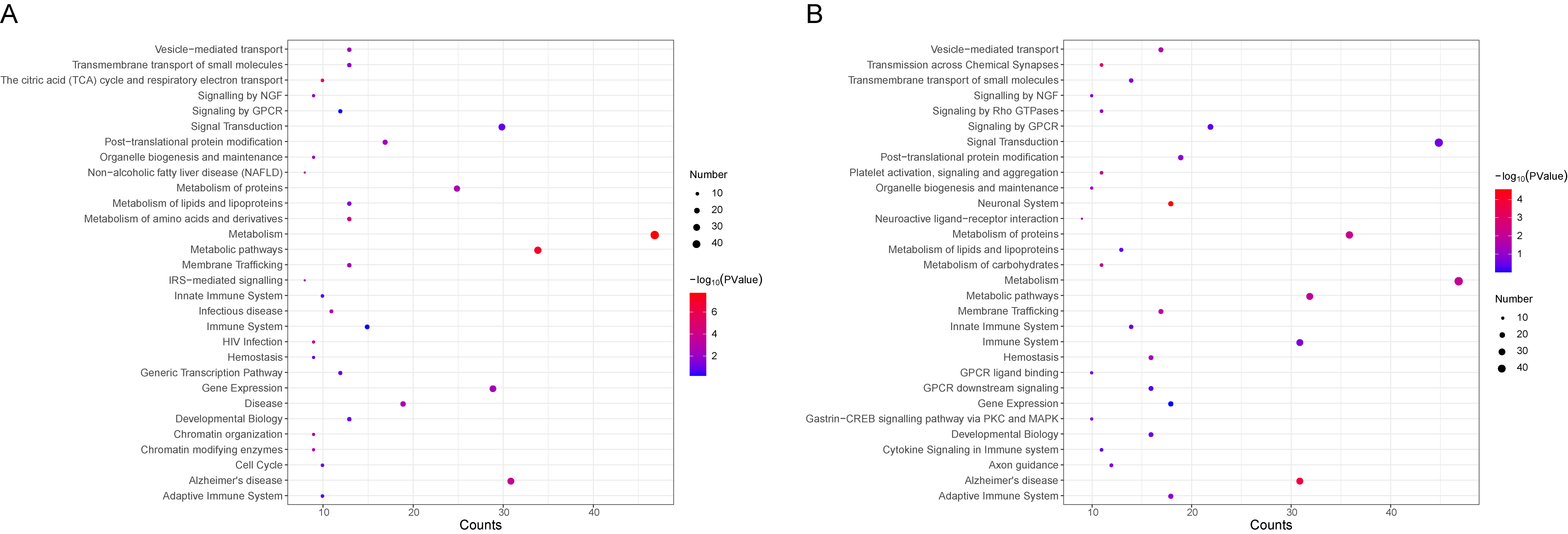

Supplement: Supplementary 1 — Figure S1: function enrichment analysis of the genes in the red (A) and yellow modules (B). The vertical axis represented the pathways, and the color alteration of the dot from red to blue indicated the alteration of P value from large to small. [file 6692974.f1.tif]

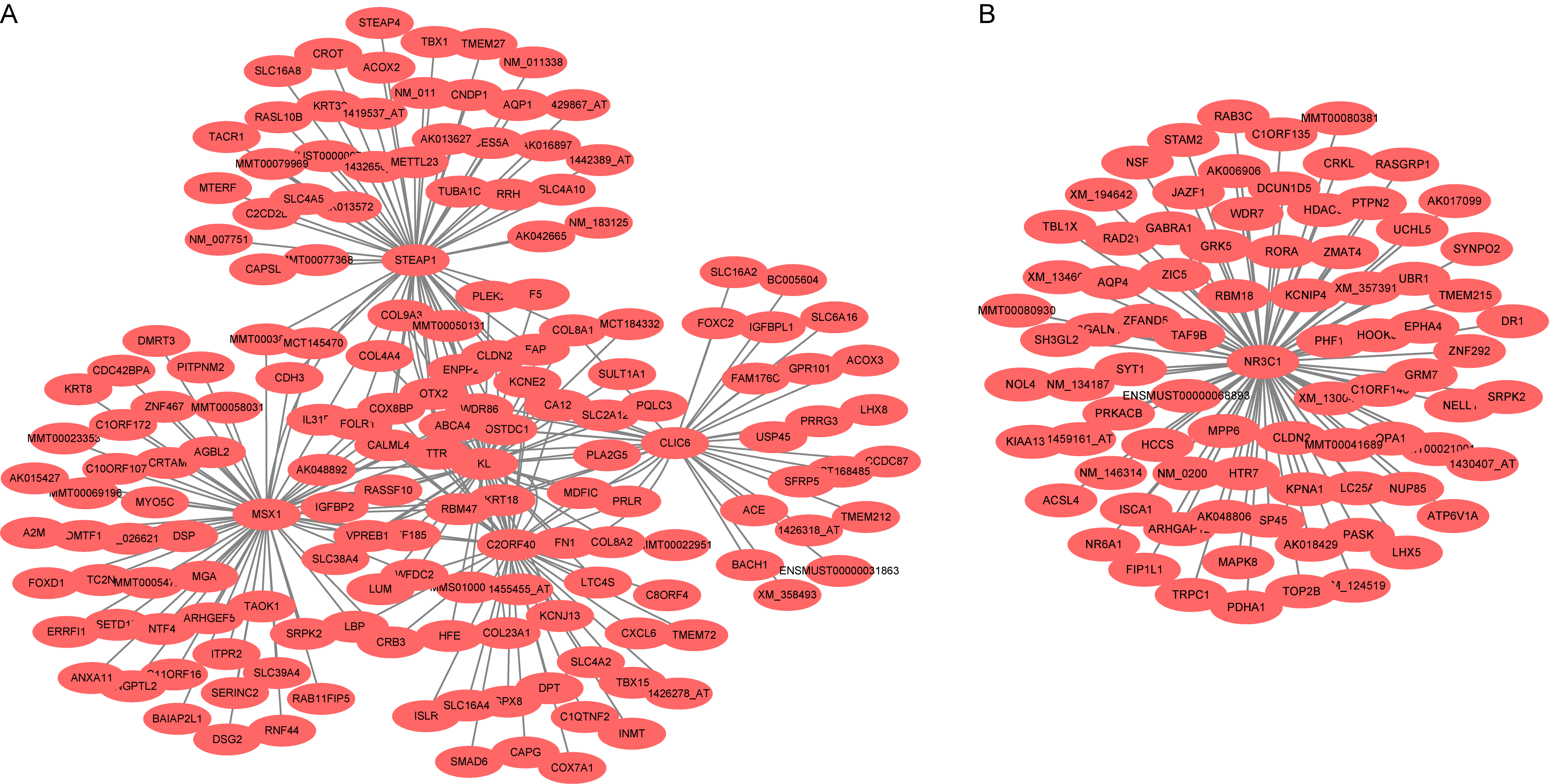

Supplement: Supplementary 2 — Figure S2: identification of the hub genes for another two of the 4 shared GCMs. (A) STEAP1, C2ORF40, MSX1, KL, and CLIC6 were the hub genes of the midnightblue module. (B) NR3C1 was the hub gene of the yellow module. [file 6692974.f2.tif]
