# Supplementary material for: Analysis of Shared Genetic Regulatory Networks for Alzheimer's Disease and Epilepsy
Source: Biomed Res Int. 2021 Oct 14;2021:6692974. doi: 10.1155/2021/6692974 (PMC8538392; doi:10.1155/2021/6692974)
Supplement: Supplementary 3 — Table S1: functional enrichment analysis of these four modules. [file 6692974.f3.docx]

Table S1. Functional enrichment analysis of these four modules.

| pathway | Database | counts | PValue |
| --- | --- | --- | --- |
| Metabolism | Reactome | 47 | 0.0082027 |
| Signal Transduction | Reactome | 45 | 0.2717478 |
| Metabolism of proteins | Reactome | 36 | 0.0052803 |
| Metabolic pathways | KEGG PATHWAY | 32 | 0.0094372 |
| Immune System | Reactome | 31 | 0.1811099 |
| Alzheimer's disease | KEGG PATHWAY | 31 | 0.0002108 |
| Signaling by GPCR | Reactome | 22 | 0.4448407 |
| Post-translational protein modification | Reactome | 19 | 0.107489 |
| Neuronal System | Reactome | 18 | 2.81E-05 |
| Adaptive Immune System | Reactome | 18 | 0.1024387 |
| Gene Expression | Reactome | 18 | 0.9866205 |
| Membrane Trafficking | Reactome | 17 | 0.0105395 |
| Vesicle-mediated transport | Reactome | 17 | 0.0183091 |
| Hemostasis | Reactome | 16 | 0.048733 |
| Developmental Biology | Reactome | 16 | 0.3267258 |
| GPCR downstream signaling | Reactome | 16 | 0.5288053 |
| Transmembrane transport of small molecules | Reactome | 14 | 0.2034175 |
| Innate Immune System | Reactome | 14 | 0.3922572 |
| Metabolism of lipids and lipoproteins | Reactome | 13 | 0.4007751 |
| Axon guidance | Reactome | 12 | 0.2094323 |
| Transmission across Chemical Synapses | Reactome | 11 | 0.0009757 |
| Platelet activation, signaling and aggregation | Reactome | 11 | 0.0084209 |
| Metabolism of carbohydrates | Reactome | 11 | 0.0086267 |
| Signaling by Rho GTPases | Reactome | 11 | 0.0956389 |
| Cytokine Signaling in Immune system | Reactome | 11 | 0.4587783 |
| Organelle biogenesis and maintenance | Reactome | 10 | 0.0608916 |
| Gastrin-CREB signalling pathway via PKC and MAPK | Reactome | 10 | 0.1720622 |
| Signalling by NGF | Reactome | 10 | 0.2089301 |
| GPCR ligand binding | Reactome | 10 | 0.2163042 |
| Neuroactive ligand-receptor interaction | KEGG PATHWAY | 9 | 0.0450395 |
